# Can reporting mood swings during oral contraceptive use predict peripartum depression? Results from the Swedish longitudinal cohort study Mom2B

**DOI:** 10.1192/j.eurpsy.2025.10135

**Published:** 2025-12-03

**Authors:** Erietta Karaviti, Hanna Wierenga, Femke Geusens, Frida Gyllenberg, Fotios C. Papadopoulos, Alkistis Skalkidou

**Affiliations:** 1 Department of Women’s and Children’s Health, Uppsala University, Uppsala, Sweden; 2 Department of Sociology, University of Tübingen, Tübingen, Germany; 3 Department of Development and Regeneration (Cluster Woman and Child), REALIFE Research Group, KU Leuven, Leuven, Belgium; 4 Department of General Practice and Primary Health Care, University of Helsinki and Helsinki University Hospital, Helsinki, Finland; 5 Department of Medical Sciences, Clinical Psychiatry, Uppsala University, Uppsala, Sweden

**Keywords:** mood swings, oral contraceptives, depression

## Abstract

**Background:**

Peripartum depression (PPD) is one of the most common pregnancy complications; nevertheless, it often goes underdiagnosed. Pinpointing important correlates is crucial for early risk identification and pathophysiology understanding. We aimed to investigate the association between self-reported mood swings during oral contraceptive (OC) use and peripartum depressive symptoms (PPDS).

**Methods:**

We used data from the Swedish longitudinal cohort study Mom2B. 3829 women who had reported previous usage of OCs were included. Self-reported mood swings during OC use were assessed through a single question, and PPDS were evaluated using established cut-offs of the Edinburgh Postnatal Depression Scale (EPDS).

**Results:**

Self-reported mood swings during OC use were associated with PPDS at gestational weeks 12–22 (OR = 1.30, 95% CI, 1.01–1.66), 24–34 (OR = 1.37, 95% CI, 1.10–1.71) and 36–42 (OR = 1.39, 95% CI, 1.05–1.82) as well as at postpartum weeks 6–13 (OR = 1.46, 95% CI, 1.12–1.92) and 24–35 (OR = 2.07, 95% CI, 1.43–2.99). Interestingly, self-reported mood swings during OC use were associated with higher odds for newly developed PPDS in early postpartum (OR for weeks 6–13 = 1.92, 95% CI: 1.19–3.08).

**Conclusions:**

Women with self-reported mood swings during OC use have higher risk of experiencing depressive symptoms across the peripartum period and twice the risk of newly developed PPDS during the early postpartum, adding to current evidence of a hormonal sensitive subgroup of women and the opportunity to use this simple question in future predictive efforts.

## Introduction

Depression is one of the most common complications of pregnancy [1]. It has been linked to heightened risk of suicide for the mother [2], sleep disturbances [3], malnutrition, decreased growth rates for the infant [4], and long term effects such as psychoemotional problems [5, 6],and low cognitive development for the child [7]. Despite the fact that about one in five mothers are depressed in the peripartum period [8, 9], the condition often goes underdiagnosed and untreated [10, 11].

Although the literature has mainly focused on the postpartum period, postpartum depression is no longer a distinct diagnosis in the Diagnostic Statistic Manual-5 (DMS-5). Instead, a depressive episode that emerges during pregnancy or during the first 4 weeks postpartum is classified as a major depressive disorder with peripartum onset [12]. ICD-11 describes peripartum depression (PPD) as a syndrome of mental and behavioral features, mostly depressive symptoms, commencing during pregnancy or within 6 weeks from delivery [13]. In research settings, this period is often extended to include the first year after delivery [14, 15].

It is considered that the sudden decrease in the levels of reproductive hormones after the delivery plays a crucial part in the pathophysiology of depression with postpartum onset [16], a mechanism that may be particularly relevant in women who have previously shown sensitivity to hormones through e.g., premenstrual syndrome (PMS), previous depression or migraine [17]. Nevertheless, studies are showing no difference in the concentrations of estrogen and progesterone between women who developed postpartum depression and women who did not [18, 19], indicating that postpartum depression does not depend solely on hormone levels, but also on women’s sensitivity to their fluctuations [20]. Similarly, ovarian hormones are found in identical levels in women with PMS and controls [21].Thus, researchers hypothesize that there is a specific subgroup of women with high sensitivity to hormonal fluctuations which can occur during different reproductive phases, who are susceptible to developing mental health symptoms during these phases [19, 22–24]. Furthermore, several studies have noted an association between postpartum depression and PMS [25–29], as well as between postpartum depression and perimenopausal depressive symptoms [25, 29, 30], suggesting that a history of a depressive episode during a hormonal transition phase might predict the occurrence of another episode during a different period of hormonal fluctuations.

Oral contraceptives (OCs) are the most common type of hormonal contraceptives in the Nordic countries [31, 32]. There are two types of OCs, progestin-only pills (POPs) and combined OCs (COCs), containing both a type of estrogen as well as a progestin. Research showcases that monophasic OCs result in hormonal fluctuations, as endogenous estradiol levels decrease during active pill phase while they dramatically increase during placebo phase [33]. Perceived mental side effects is one of the reasons why women discontinue the usage of OCs in Sweden [34], affecting an estimated 4–10% of users [35]. However, a Swedish register based study showed no direct association between COCs and depression [36]. Additionally, a recent meta-analysis concludes no increase in depressive symptoms in adult users of hormonal contraceptives [37]. Nevertheless, a randomized controlled trial noted a causal effect of COCs on depressed mood [38], while two Danish studies showcase that the use of hormonal contraceptives, including OCs, is associated with an increased risk of depression especially when initiated in adolescence [39] or postpartum [40]. Interestingly, a randomized controlled trial noted that COCs might cause mood changes such as mood swings in the intermenstrual phase, while in the premenstrual phase they were associated with improved mood [41].

Research on the association between mood disturbances caused by hormonal contraceptives and PPD is scarce and has mixed results. First, women who had experienced both PMS and postpartum depression exhibited a higher likelihood of experiencing side effects from OCs compared to women who had experienced only postpartum depression [42]. Then, other studies have also identified an association between mood symptoms from hormonal contraceptives and postpartum mood disorders [28, 43]. Meanwhile, a recent study reported no significant association between mood swings from OCs and postpartum depression [44].

Understanding the predictors and correlates of PPD is crucial, as it can pave the way for high-risk identification, earlier diagnosis and intervention, particularly since PPD is commonly underdiagnosed. This can further advance our understanding of PPD pathophysiology and its possible subtypes [17, 45]. Aiming to address the gap in the existing literature we seek to present a variable of potential interest for future PPD prediction models. Thus, this study examined whether women with self-reported mood swings during OC use had higher risk of reporting depressive symptoms at different time points during the peripartum period compared to women without such experiences. We furthermore examined the association between self-reported mood swings during OC use in relation to newly developed peripartum depressive symptoms (PPDS) in different time points in the peripartum period.

## Methods

The present study uses data from Mom2B study, a Swedish national longitudinal cohort study focusing on the development of prediction models for peripartum complications that utilizes a smartphone application for data collection [46]. Mom2B study fulfills the General Data Protection Regulation (GDPR) requirements and the ethical approval was granted from the Swedish Ethical Review Authority (dnr: 2019/01170, with amendments).

### Study population

All adult Swedish-speaking women, pregnant or within 3 months postpartum, were eligible to participate. Recruitment was conducted using social media campaigns, alongside posters and brochures handed out in primary and maternal healthcare centers throughout Sweden. The recruitment process started when the Mom2B application was launched in November 2019 and is still ongoing. Each participant got information about the aims of the study, the confidentiality and security of their data before giving consent and registering. Participants could withdraw consent at any time without the need to provide a reason.

**Figure 1.**
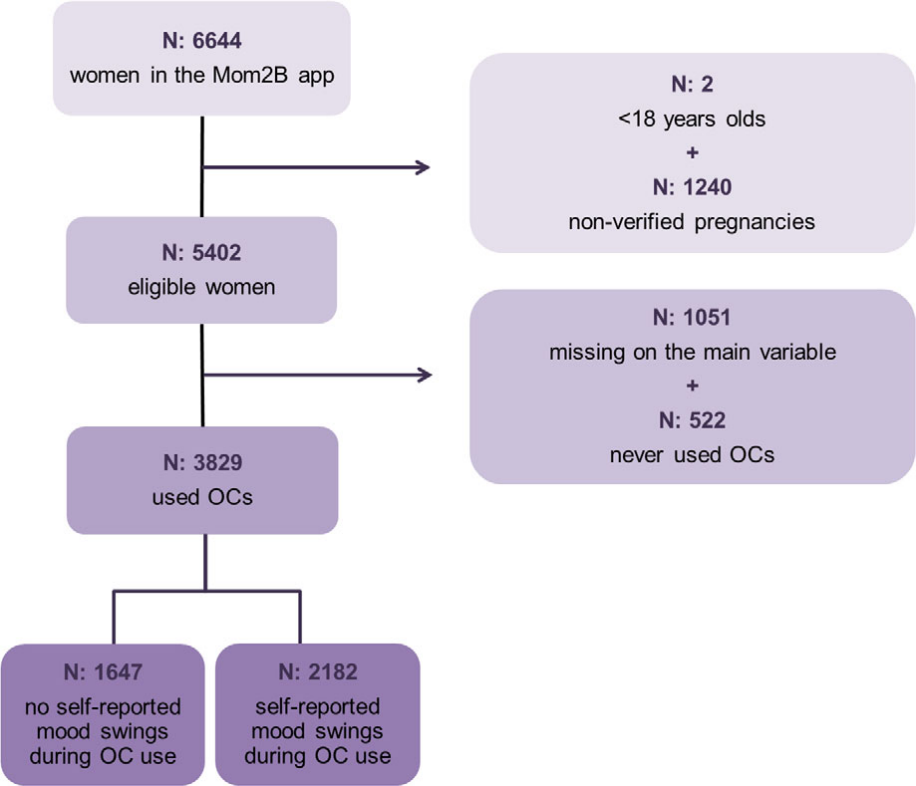
Flowchart. OCs, oral contraceptives.

For the present study, we analyzed data collected between November 2019 and December 2024, including all women enrolled in the study thus far (n = 6644). As shown in Figure 1, nonverified pregnancies by the pregnancy registry, women who did not answer to the question about OCs and women who had never taken OCs were excluded from the analysis. Consequently, the sample used for analysis consisted of 3829 women.

### Exposure

To enhance generalizability, we included women with milder mood fluctuations, not only those with a clinical depression diagnosis while on OCs; thus, we chose our exposure to be self-reported mood swings during OC use which was assessed with the question: “Have you had mood swings from birth control pills?.” Women could choose between the following answers: “No,” “Yes” and “Have never used birth control pills.”

### Outcome and covariates

The outcome of interest was PPDS. Depressive symptoms were evaluated using the Edinburgh Postnatal Depression Scale (EPDS). EPDS is a 10-item self-report questionnaire made for postpartum depression screening [47]. Depending on the symptom severity, each response is scored from 0 to 3; therefore, total EPDS scores vary from 0 to 30. A cut-off of 13 was used for pregnant women, while a cut-off of 12 was used for postpartum women, in line with the Swedish validation of EPDS for pregnancy and postpartum stages, respectively [48,49]. EPDS was administered via the Mom2B application. For our analysis, we utilized EPDS scores from six different time points (gestational weeks 12–22, 24–34, 36–42 and postpartum weeks 6–13, 14–23, and 24–35).

The confounders taken into account were age at registration, BMI before pregnancy, highest level of education completed, history of depression, and medical indications for the use of OCs including any among the following: PMS, polycystic ovary syndrome (PCOS) and endometriosis.

### Statistical analyses

Demographic and clinical characteristics (including PMS, PCOS, and endometriosis) were compared between exposure groups using Pearson’s chi-square test. In order to compare the distributions of the total EPDS score on each time point between the two groups, we used Mann–Whitney *U* Test for each of the six different time points, as the EPDS scores were not normally distributed. We evaluated the differences in rates of those above the EPDS cut-off score at each time point in relation to self-reported mood swings during OC use using Pearson’s chi-square test for each of the six time points. In order to focus on the newly developed PPDS, we ran the same analysis excluding women having EPDS scores above the cut-off on the previous time point. This analysis was applied to all time points, except the first pregnancy time point as we do not have an EPDS score prior to gestational week 12, thus making it impossible to exclude women with previous EPDS scores above the cut-off.

To estimate the association between self-reported mood swings and PPDS, we conducted logistic regressions at all time points for each covariate individually and for the full multivariable model. The outcome was dichotomized EPDS; predictors were self-reported mood swings and confounders. To examine the association with newly developed PPDS, we ran logistic regressions at all time points except the first one, excluding those who already had EPDS above the cut-off. Each regression was performed both as a crude model and as an adjusted model including the confounders mentioned above as predictors covariates. Missing data were accounted for via multiple imputation with 50 imputed datasets.

SPSS version 29.0 was used for the statistical analyses. Statistical significance was set at a p value <0.05.

## Results

Among the 3829 women who had used OCs, 2182 (57%) reported experiencing mood swings. Table 1 showcases demographics and clinical characteristics among the two exposure groups. Women with self-reported mood swings during OC use had higher rates of endometriosis (p = 0.010) and PMS (p < 0.001). Additionally, they had lower or higher than normal BMI before pregnancy (p = 0.010), lower levels of education (p < 0.001), and higher rates of smoking the last three months before pregnancy (p = 0.001) in comparison to women with no self-reported mood swings.

**Table 1.** Demographics and clinical characteristics of the participants by groups; no self-reported mood swings during OC use and self-reported mood swings during OC use

	Variable	N	No self-reported mood swings during OC use (N = 1647)	Self-reported mood swings during OC use (N = 2182)	p value
Age, years	18–25	317	103 (6.3%)	214 (9.8%)	<0.001*
	26–30	1355	539 (32.7%)	816 (37.4%)	
	31–35	1519	680 (41.3%)	839 (38.5%)	
	>35	638	325 (19.7%)	313 (14.3%)	
BMI before pregnancy, kg/m^2^	<18.5	80	24 (1.5%)	56 (2.7%)	0.010*
	18.5- < 25	1951	879 (56.2%)	1072 (51.6%)	
	25- < 30	1027	426 (27.2%)	601 (28.9%)	
	≥30	583	236 (15.1%)	347 (16.7%)	
	Missing	188	-	-	
Education	Not gone to school	1	0	1	<0.001*
	Primary school	75	23 (1.4%)	52 (2.5%)	
	High school	677	243 (15.3%)	434 (20.5%)	
	Polytechnic/vocational training	360	139 (8.7%)	221 (10.4%)	
	University /college	2594	1185 (74.5%)	1409 (66.6%)	
	Missing	122	-	-	
Relationship status	Single	75	34 (2.1%)	41 (1.9%)	0.878
	In relationship	75	31 (2.0%)	44 (2.1%)	
	Cohabiting	3555	1522 (95.9%)	2033 (96.0%)	
	Missing	124	-	-	
Smoking 3 months before pregnancy	Yes	476	171 (11.2%)	305 (15.0%)	0.001*
	Missing	268	-	-	
PMS	Yes	2376	790 (48.1%)	1586 (73.0%)	<0.001*
	Missing	13	-	-	
PCOS	Yes	286	118 (7.2%)	168 (7.7%)	0.530
	Missing	6	-	-	
Endometriosis	Yes	168	56 (3.6%)	112 (5.4%)	0.010*
	Missing	172	-	-	
Parity	0	1752	798 (51.3%)	954 (46.1%)	<0.001*
	1	1313	563 (36.2%)	750 (36.2%)	
	≥2	560	194 (12.5%)	366 (17.7%)	
	Missing	204	-	-	

BMI, body mass index; OCs, oral contraceptives; PCOS, polycystic ovary syndrome; PMS, premenstrual syndrome. *p<0.05

**Figure 2.**
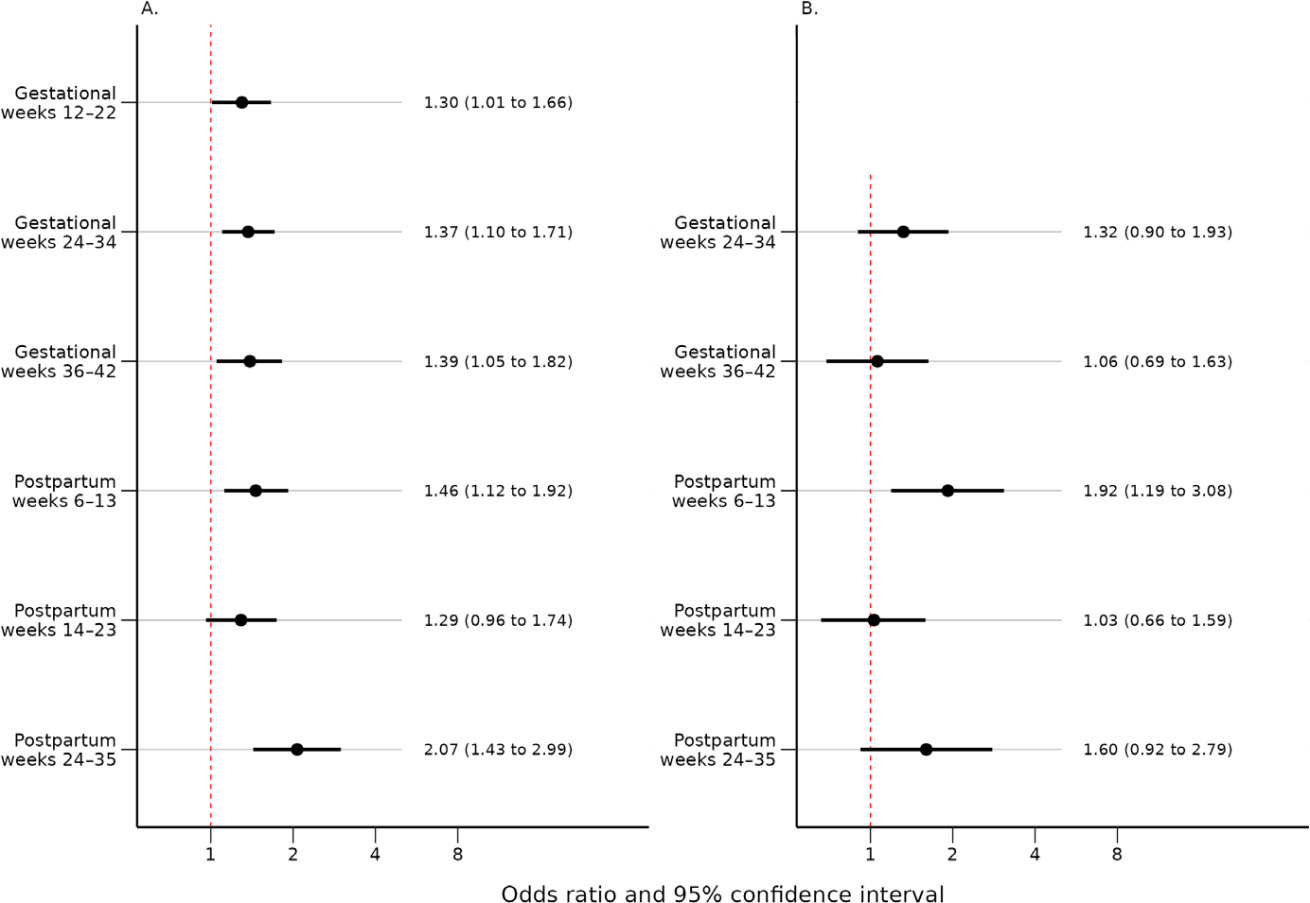
(A) Odds ratios and 95% confidence intervals for the association between self-reported mood swings during OC use and an EPDS score above cut-off for each time point adjusted for age, BMI, education, medical indications for OCs, and history of depression. (B) Odds ratios and 95% confidence intervals for the association between self-reported mood swings during OC use and an EPDS score above cut-off for each time point, after exclusion of women with high EPDS scores on the previous time point (≥13 for the gestational time points and ≥ 12 for the postpartum time points). Adjusted for age, BMI, education, medical indications for OCs, and history of depression. BMI, body mass index; EPDS, Edinburgh Postnatal Depression Scale; OCs, oral contraceptives.

Women with self-reported mood swings during OC use had statistically significant higher median EPDS total score at every time point throughout pregnancy and postpartum compared to women who had not (p < 0.001) (Table 2). Self-reported mood swings during OC use were associated with dichotomized EPDS scores in every pregnancy and postpartum time point (p < 0.001) (Table 3). After excluding women having EPDS scores above the cut-off on the previous time point, the association remained significant only in postpartum weeks 6–13 (p < 0.001) and 24–35 (p = 0.039) (Table 4). The adjusted logistic regressions confirmed the patterns observed above. Self-reported mood swings during OC use were associated with higher odds of scoring above the cut-off on EPDS in all pregnancy (ORs between 1.30 and 1.39) and postpartum (ORs between 1.46 and 2.07) time points, except for postpartum 14–23 (Figure 2A). After excluding women having EPDS scores above the cut-off on the previous time point, self-reported mood swings during OC use were associated with higher odds of newly developed PPDS only in postpartum weeks 6–13, with an OR of 1.92 (95% CI, 1.19–3.08) (Figure 2B).

**Table 2.** Median EPDS scores and IQR during pregnancy and postpartum by groups: no self-reported mood swings during OC use and self-reported mood swings during OC use

EPDS time point	N (missing)	No self-reported mood swings during OC use (N = 1647)		Self-reported mood swings during OC use (N = 2182)		p value
		Median	IQR	Median	IQR	
Gestational weeks 12–22	2126 (1703)	6	3–10	7	4–12	<0.001*
Gestational weeks 24–34	2442 (1387)	6	3–10	7	4–12	<0.001*
Gestational weeks 36–42	1982 (1847)	5	2–9	7	4–11	<0.001*
Postpartum weeks 6–13	1695 (2134)	5	2–8.25	7	3–11	<0.001*
Postpartum weeks 14–23	1359 (2470)	5	2–9	6	3–11	<0.001*
Postpartum weeks 24–35	1097 (2732)	4	2–8	6	3–10.50	<0.001*

EPDS, Edinburgh Postnatal Depression Scale; IQR, interquartile range; OCs, oral contraceptives. *p<0.05

**Table 3.** Number of women and percentages for normal and high EPDS throughout pregnancy and postpartum by groups: no self-reported mood swings during OC use and self-reported mood swings during OC use

EPDS time point	Dichotomized score	N	No self-reported mood swings during OCs use (N = 1647)	Self-reported mood swings during OC use (N = 2182)	p value
Gestational weeks 12–22	0–12	1736	794 (85.5%)	942 (78.4%)	<0.001*
	13–30	390	131 (14.2%)	259 (21.6%)	
	Missing	1705	-	-	
Gestational weeks 24–34	0–12	1967	899 (84.9%)	1068 (77.2%)	<0.001*
	13–30	475	160 (15.1%)	315 (22.8%)	
	Missing	1387	-	-	
Gestational weeks 36–42	0–12	1680	750 (88.8%)	930 (81.8%)	<0.001*
	13–30	302	95 (11.2%)	207 (18.2%)	
	Missing	1847	-	-	
Postpartum weeks 6–13	0–11	1381	633 (86.2%)	748 (77.8%)	<0.001*
	12–30	314	101 (13.8%)	213 (22.2%)	
	Missing	2134	-	-	
Postpartum weeks 14–23	0–11	1106	529 (85.5%)	577 (78.0%)	<0.001*
	12–30	253	90 (14.5%)	163 (22.0%)	
	Missing	2470	-	-	
Postpartum weeks 24–35	0–11	920	447 (90.1%)	473 (78.7%)	<0.001*
	12–30	177	49 (9.9%)	128 (21.3%)	
	Missing	2732	-	-	

EPDS, Edinburgh Postnatal Depression Scale; OCs, oral contraceptives. *p<0.05

**Table 4.** Number of women and percentages for normal and high EPDS throughout pregnancy and postpartum by groups: no self-reported mood swings during OC use and self-reported mood swings during OC use, after exclusion of women with high EPDS scores on the previous time point (≥13 for the gestational time points and ≥ 12 for the postpartum time points)

EPDS time point	Dichotomized score	N	No self-reported mood swings during OC use (N = 1647)	Self-reported mood swings during OC use (N = 2182)	p value
Gestational weeks 24–34	0–12	1126	532 (91.1%)	594 (87.7%)	0.055
	13–30	135	52 (8.9%)	83 (12.3%)	
	Missing	475	-	-	
Gestational weeks 36–42	0–12	1265	582 (93.6%)	683 (91.3%)	0.118
	13–30	105	40 (6.4%)	65 (8.7%)	
	Missing	597	-	-	
Postpartum weeks 6–13	0–11	995	462 (94.5%)	533 (88.4%)	<0.001*
	12–30	97	27 (5.5%)	70 (11.6%)	
	Missing	295	-	-	
Postpartum weeks 14–23	0–11	937	454 (91.5%)	483 (89.4%)	0.254
	12–30	99	42 (8.5%)	57 (10.6%)	
	Missing	345	-	-	
Postpartum weeks 24–35	0–11	760	375 (94.2%)	385 (90.4%)	0.039*
	12–30	64	23 (5.8%)	41 (9.6%)	
	Missing	282	-	-	

EPDS, Edinburgh Postnatal Depression Scale; OCs, oral contraceptives. *p<0.05

## Discussion

More than half of the OC users in our study reported mood swings during OC use. Although higher than expected, mental side effects have been shown to be the most commonly reported adverse effects and a leading cause of discontinuation of hormonal contraception [50, 51].

The results of our study point toward a robust association between self-reported mood swings during OC use and the presence of significant depressive symptoms throughout all of pregnancy and most of the postpartum time points. Interestingly, another major finding of our study was that women with self-reported mood swings during OC use have twice the risk of newly developed PPDS during postpartum weeks 6–13.

Few studies have explored this association throughout pregnancy and the postpartum period. Our results are in line with a previous study that noted that history of mood swings from OCs was a significant risk factor for mood disorders during postpartum weeks 6–8 [25]. Furthermore, a recent Danish cohort study researching hormonal contraceptives showcased a link between history of depression associated with hormonal contraceptives and postpartum depression. The association was maintained even when including those depressed during pregnancy [39].

Although it is unclear whether OCs create a hypo- or hyper-hormonal state in the brain [52], OC users’ endogenous estradiol and progesterone levels are lower than among naturally cycling women [53, 54] and the levels of inflammatory markers higher [55, 56]. In parallel, women after delivery present with decreased levels of estradiol and progesterone [57, 58], while some inflammation markers rise and are even found to be increased in those with postpartum depression [59], indicating a possible common pathophysiological mechanism. There might of course be other mechanisms by which OCs impact on mood, such as higher levels of estrogen receptor stimulation, the choice of synthetic progestogen, or the lower androgen levels. It cannot be ruled out that depressive symptoms during pregnancy could be due to the first of these mechanisms, while peripartum depressive symptoms to the latter.

Our findings pinpoint strongest associations during the early postpartum and late postpartum time points. In the early postpartum, estrogens and progesterone suddenly drop [58], while oxytocin rises [60]. Additionally, the last postpartum time point could be associated with the ending of breastfeeding and thus drop in oxytocin and prolactin, or even the start of hormonal contraception [60]. As a result, both periods represent phases of great hormonal fluctuations, leaving many women vulnerable to mood disturbances. Bloch et al. pharmacologically imitated the fluctuations of sex hormones that normally occur during pregnancy and postpartum on euthymic women and found that, in those with a history of postpartum depression, depressive symptoms increased when estrogen and progesterone were introduced and peaked during the phase when both hormones were withdrawn [18]. This aligns especially with our finding that women with self-reported mood swings during OC use had a higher risk of newly developed PPDS during postpartum weeks 6–13. It is also in line with another Mom2B substudy showcasing that women with PMS exhibited higher risk of developing PPD during all the postpartum time points [61]. These findings indicate that a certain subgroup of women have an abnormal response to the abrupt decrease of sex hormones following delivery and present more evidence for the existence of a hormonal sensitive subgroup of women to a growing body of literature.

The biological mechanism behind this hormonal sensitivity is yet to be fully understood. Some researchers suggest that it might be attributed to a dysregulation of estrogen signaling as most of the116 transcripts that were found to be differentially expressed between women with postpartum depression and euthymic women in late pregnancy were linked to it [19]. Another study showed that women who developed postpartum depression exhibited attenuated connectivity in the anterior cingulate cortex, amygdala, hippocampus, and dorsolateral prefrontal cortex despite having similar derivatives of progesterone levels to healthy comparison subjects [62]. Although women with and without postpartum depression exhibit comparable levels of allopregnanolone, this metabolite of progesterone has been associated with the pathophysiology of reproductive mood disorders such as PMDD and PPD by inducing affective dysregulation in a susceptible group of women [20, 63]. Clinical trials have also highlighted the robust antidepressant effect of Brexanolone, a synthetic allopregnanolone analog [64], which is now an FDA-approved treatment of postpartum depression [65].

### Clinical implications and future studies

Our results indicate that self-reported mood swings during OC use is a strong risk factor for PPD. Although causality cannot be assumed, this finding should be regarded as an indicator worth considering in clinical assessments. Incorporating a brief question about mood disturbances during OC use into medical intake during the early stages of pregnancy could help identify high-risk women, ensuring earlier diagnosis and intervention for women at high risk of developing PPD.

Future studies could investigate if mood deterioration after the use of specific types of OCs is more strongly associated with PPD. Categorizing participants based on OC type yields greater precision in assessing the relationship between mood disturbances from OCs and PPD. Additionally, other hormonal contraceptives, such as intrauterine devices (IUDs) or injections, should be investigated separately. Finally, it would be interesting for future studies to examine the association between mood swings during OCs and depressive symptoms during other periods of significant hormonal fluctuations, such as menarche or perimenopause.

### Strengths and limitations

The study used data from the national Mom2B project, a Swedish longitudinal study that is being conducted via a smart phone application, which enables the close monitoring of a national, population-based cohort of participants from pregnancy to up to 1 year postpartum.

The use of validated instruments for the assessment of depressive symptoms and controlling for several confounding factors are strengths of the present work. The replication of known associations, such as with history of depression, high BMI, and medical indications for OCs (full tables can be found in the Supplementary Material) is also seen as a strength, as they are in line with previous studies [66–68]. A novelty of the current work is the subanalysis that assessed the newly developed PPDS at each peripartum time point, showcasing the early postpartum one as a period of particular vulnerability for women with self-reported mood swings during OC use.

Limitations should also be considered when interpreting our findings. Firstly, as the Mom2B application is only available in Swedish, our cohort mainly consists of women born in Sweden, influencing the generalizability of our results. Additionally, the study’s focus on perinatal mental health may have attracted women with prior mental health issues. Moreover, it should be noted that there is a considerable amount of missing data relating to the EPDS questionnaire in the last postpartum time points. This may be due to some participants not having reached that stage of postpartum alongside time constraints and survey fatigue [69]. To address this problem, we used 50 multiple imputations to handle the missing in each time point. Furthermore, the specific type of OCs that each woman has used (that is, POPs or COCs) was not specified in the related surveys. The heterogeneity of OCs makes it hard to draw more precise conclusions. Lastly, mood swings were evaluated based on a self-report question that was asked during pregnancy. Thus, we can only capture whether OC users perceive the mood swings to be caused by the contraceptive method – not whether there is an actual causal relationship.

## Conclusions

In summary, this Mom2B substudy showcases that women with self-reported mood swings during OC use had higher risk of depressive symptoms across the peripartum period, but also notably increased risk of newly developed PPDS during the early postpartum. Our findings contribute to the growing body of evidence indicating a hormonal sensitive subgroup of women who struggle in their response to hormonal fluctuations and suggest that self-reported mood swings during OC use could be used when predicting and identifying high-risk women. Furthermore, our work highlights the importance of incorporating questions about each woman’s experience with OCs in the routine check-ups during early pregnancy.

## Supporting information

10.1192/j.eurpsy.2025.10135.sm001 Karaviti et al. supplementary material Karaviti et al. supplementary material

## Data Availability

The data supporting this research are available from the authors on a reasonable request.

## Supplementary material

The supplementary material for this article can be found at http://doi.org/10.1192/j.eurpsy.2025.10135.
