# Supplementary material for: Can reporting mood swings during oral contraceptive use predict peripartum depression? Results from the Swedish longitudinal cohort study Mom2B
Source: Eur Psychiatry. 2025 Dec 3;69(1):e4. doi: 10.1192/j.eurpsy.2025.10135 (PMC12816930; doi:10.1192/j.eurpsy.2025.10135)
Supplement: Karaviti et al. supplementary material [file S0924933825101351sup001.zip › S0924933825101351sup003.docx]

|  | Adjusted | Adjusted |
| --- | --- | --- |
| **Variables** | **Odds ratio (95% CI)** | **p value** |
| **Self-reported mood swings** | 1.03 (0.66 – 1.59) | 0.905 |
| **Age** | 0.95 (0.90 – 1.01) | 0.079 |
| **BMI** |  |  |
| **Low / Normal BMI** | Reference | - |
| **High BMI** | 1.82 (1.18 – 2.80) | **0.007** |
| **Education** |  |  |
| **No university** | 1.09 (0.65 – 1.81) | 0.741 |
| **University** | Reference | - |
| **Medical indications for OCs** | 1.75 (1.06 – 2.89) | **0.027** |
| **History of depression** | 1.41 (1.02 – 1.94) | **0.038** |
