# Supplementary material for: Can reporting mood swings during oral contraceptive use predict peripartum depression? Results from the Swedish longitudinal cohort study Mom2B
Source: Eur Psychiatry. 2025 Dec 3;69(1):e4. doi: 10.1192/j.eurpsy.2025.10135 (PMC12816930; doi:10.1192/j.eurpsy.2025.10135)
Supplement: Karaviti et al. supplementary material [file S0924933825101351sup001.zip › S0924933825101351sup005.docx]

|  | Adjusted | Adjusted |
| --- | --- | --- |
| **Variables** | **Odds ratio (95% CI)** | **p value** |
| **Self-reported mood swings** | 1.60 (0.92 – 2.79) | 0.097 |
| **Age** | 0.99 (0.92 – 1.05) | 0.690 |
| **BMI** |  |  |
| **Low / Normal BMI** | Reference | - |
| **High BMI** | 0.94 (0.55 – 1.61) | 0.829 |
| **Education** |  |  |
| **No university** | 0.88 (0.45 – 1.73) | 0.718 |
| **University** | Reference | - |
| **Medical indications for OCs** | 0.96 (0.55 – 1.69) | 0.899 |
| **History of depression** | 1.48 (1.02 – 2.17) | **0.041** |
