# Supplementary material for: Can reporting mood swings during oral contraceptive use predict peripartum depression? Results from the Swedish longitudinal cohort study Mom2B
Source: Eur Psychiatry. 2025 Dec 3;69(1):e4. doi: 10.1192/j.eurpsy.2025.10135 (PMC12816930; doi:10.1192/j.eurpsy.2025.10135)
Supplement: Karaviti et al. supplementary material [file S0924933825101351sup001.zip › S0924933825101351sup007.docx]

|  | Adjusted | Adjusted |
| --- | --- | --- |
| **Variables** | **Odds ratio (95% CI)** | **p value** |
| **Self-reported mood swings** | 1.30 (1.01 – 1.66) | **0.037** |
| **Age** | 0.99 (0.96 – 1.02) | 0.407 |
| **BMI** |  |  |
| **Low BMI** | 1.42 (0.72 – 2.81) | 0.312 |
| **Normal BMI** | Reference | - |
| **High BMI** | 0.99 (0.78 – 1.26) | 0.961 |
| **Education** |  |  |
| **No school/ just primary or high school** | 1.78 (1.34 – 2.38) | **<0.001** |
| **Polytechnic or Vocational training** | 1.52 (1.06 – 2.20) | **0.024** |
| **University** | Reference | **-** |
| **Medical indications for OCs** | 1.41 (1.08 – 1.84) | **0.010** |
| **History of depression** | 2.05 (1.72 – 2.44) | **<0.001** |
