# Supplementary material for: Can reporting mood swings during oral contraceptive use predict peripartum depression? Results from the Swedish longitudinal cohort study Mom2B
Source: Eur Psychiatry. 2025 Dec 3;69(1):e4. doi: 10.1192/j.eurpsy.2025.10135 (PMC12816930; doi:10.1192/j.eurpsy.2025.10135)
Supplement: Karaviti et al. supplementary material [file S0924933825101351sup001.zip › S0924933825101351sup008.docx]

|  | Unadjusted analysis | Unadjusted |
| --- | --- | --- |
| **Variables** | **Odds ratio (95% CI)** | **p value** |
| **Self-reported mood swings** | 1.66 (1.34 – 2.04) | **<0.001** |
