# Supplementary material for: Can reporting mood swings during oral contraceptive use predict peripartum depression? Results from the Swedish longitudinal cohort study Mom2B
Source: Eur Psychiatry. 2025 Dec 3;69(1):e4. doi: 10.1192/j.eurpsy.2025.10135 (PMC12816930; doi:10.1192/j.eurpsy.2025.10135)
Supplement: Karaviti et al. supplementary material [file S0924933825101351sup001.zip › S0924933825101351sup009.docx]

|  | Adjusted | Adjusted |
| --- | --- | --- |
| **Variables** | **Odds ratio (95% CI)** | **p value** |
| **Self-reported mood swings** | 1.37 (1.10 – 1.71) | **0.005** |
| **Age** | 0.99 (0.96 – 1.01) | 0.295 |
| **BMI** |  |  |
| **Low BMI** | 0.92 (0.44 – 1.90) | 0.819 |
| **Normal BMI** | Reference | - |
| **High BMI** | 1.18 (0.95 – 1.46) | 0.133 |
| **Education** |  |  |
| **No school/ just primary or high school** | 1.26 (0.96 – 1.64) | 0.097 |
| **Polytechnic or Vocational training** | 1.32 (0.94 – 1.85) | 0.104 |
| **University** | Reference | **-** |
| **Medical indications for OCs** | 1.29 (1.02 – 1.63) | **0.031** |
| **History of depression** | 1.64 (1.40 – 1.92) | **<0.001** |
