# Supplementary material for: Can reporting mood swings during oral contraceptive use predict peripartum depression? Results from the Swedish longitudinal cohort study Mom2B
Source: Eur Psychiatry. 2025 Dec 3;69(1):e4. doi: 10.1192/j.eurpsy.2025.10135 (PMC12816930; doi:10.1192/j.eurpsy.2025.10135)
Supplement: Karaviti et al. supplementary material [file S0924933825101351sup001.zip › S0924933825101351sup011.docx]

|  | Adjusted | Adjusted |
| --- | --- | --- |
| **Variables** | **Odds ratio (95% CI)** | **p value** |
| **Self-reported mood swings** | 1.39 (1.05 – 1.82) | **0.020** |
| **Age** | 1.00 (0.97 – 1.03) | 0.810 |
| **BMI** |  |  |
| **Low BMI** | 1.08 (0.43 – 2.71) | 0.869 |
| **Normal BMI** | Reference | - |
| **High BMI** | 1.00 (0.77 – 1.30) | 0.984 |
| **Education** |  |  |
| **No school/ just primary or high school** | 1.51 (1.08 – 2.1009) | **0.015** |
| **Polytechnic or Vocational training** | 1.49 (0.99 – 2.23) | 0.054 |
| **University** | Reference | - |
| **Medical indications for OCs** | 1.43 (1.06 – 1.93) | **0.017** |
| **History of depression** | 1.92 (1.58 – 2.33) | **<0.001** |
