# Supplementary material for: Can reporting mood swings during oral contraceptive use predict peripartum depression? Results from the Swedish longitudinal cohort study Mom2B
Source: Eur Psychiatry. 2025 Dec 3;69(1):e4. doi: 10.1192/j.eurpsy.2025.10135 (PMC12816930; doi:10.1192/j.eurpsy.2025.10135)
Supplement: Karaviti et al. supplementary material [file S0924933825101351sup001.zip › S0924933825101351sup013.docx]

|  | Adjusted | Adjusted |
| --- | --- | --- |
| **Variables** | **Odds ratio (95% CI)** | **p value** |
| **Self-reported mood swings** | 1.46 (1.12 – 1.92) | **0.006** |
| **Age** | 1.00 (0.96 – 1.03) | 0.813 |
| **BMI** |  |  |
| **Low BMI** | 0.56 (0.13 – 2.48) | 0.447 |
| **Normal BMI** | Reference | - |
| **High BMI** | 1.16 (0.89 – 1.51) | 0.266 |
| **Education** |  |  |
| **No school/ just primary or high school** | 1.42 (0.99 – 2.02) | 0.054 |
| **Polytechnic or Vocational training** | 1.72 (1.14 – 2.58) | **0.009** |
| **University** | Reference | - |
| **Medical indications for OCs** | 1.52 (1.13 – 2.03) | **0.005** |
| **History of depression** | 1.75 (1.44 – 2.13) | **<0.001** |
