# Supplementary material for: Can reporting mood swings during oral contraceptive use predict peripartum depression? Results from the Swedish longitudinal cohort study Mom2B
Source: Eur Psychiatry. 2025 Dec 3;69(1):e4. doi: 10.1192/j.eurpsy.2025.10135 (PMC12816930; doi:10.1192/j.eurpsy.2025.10135)
Supplement: Karaviti et al. supplementary material [file S0924933825101351sup001.zip › S0924933825101351sup015.docx]

|  | Adjusted | Adjusted |
| --- | --- | --- |
| **Variables** | **Odds ratio (95% CI)** | **p value** |
| **Self-reported mood swings** | 1.29 (0.96 – 1.74) | 0.094 |
| **Age** | 0.98 (0.94 – 1.01) | 0.187 |
| **BMI** |  |  |
| **Low BMI** | 1.58 (0.60 – 4.21) | 0.356 |
| **Normal BMI** | Reference | - |
| **High BMI** | 1.44 (1.07 – 1.93) | **0.015** |
| **Education** |  |  |
| **No school/ just primary or high school** | 1.52 (1.03 – 2.25) | **0.034** |
| **Polytechnic or Vocational training** | 1.08 (0.66 – 1.76) | 0.766 |
| **University** | Reference | - |
| **Medical indications for OCs** | 1.60 (1.15 – 2.23) | **0.005** |
| **History of depression** | 1.63 (1.31 – 2.02) | **<0.001** |
