# Supplementary material for: Can reporting mood swings during oral contraceptive use predict peripartum depression? Results from the Swedish longitudinal cohort study Mom2B
Source: Eur Psychiatry. 2025 Dec 3;69(1):e4. doi: 10.1192/j.eurpsy.2025.10135 (PMC12816930; doi:10.1192/j.eurpsy.2025.10135)
Supplement: Karaviti et al. supplementary material [file S0924933825101351sup001.zip › S0924933825101351sup017.docx]

|  | Adjusted | Adjusted |
| --- | --- | --- |
| **Variables** | **Odds ratio (95% CI)** | **p value** |
| **Self-reported mood swings** | 2.07 (1.43 – 2.99) | **<0.001** |
| **Age** | 0.99 (0.96 – 1.04) | 0.813 |
| **BMI** |  |  |
| **Low BMI** | 0.93 (0.22 – 3.88) | 0.926 |
| **Normal BMI** | Reference | - |
| **High BMI** | 1.23 (0.87 – 1.74) | 0.231 |
| **Education** |  |  |
| **No school/ just primary or high school** | 1.65 (1.04 – 2.63) | **0.034** |
| **Polytechnic or Vocational training** | 1.07 (0.60 – 1.93) | 0.810 |
| **University** | Reference | - |
| **Medical indications for OCs** | 1.42 (0.97 – 2.08) | 0.074 |
| **History of depression** | 1.43 (1.11 – 1.85) | **0.005** |
