# Supplementary material for: Can reporting mood swings during oral contraceptive use predict peripartum depression? Results from the Swedish longitudinal cohort study Mom2B
Source: Eur Psychiatry. 2025 Dec 3;69(1):e4. doi: 10.1192/j.eurpsy.2025.10135 (PMC12816930; doi:10.1192/j.eurpsy.2025.10135)
Supplement: Karaviti et al. supplementary material [file S0924933825101351sup001.zip › S0924933825101351sup019.docx]

|  | Adjusted | Adjusted |
| --- | --- | --- |
| **Variables** | **Odds ratio (95% CI)** | **p value** |
| **Self-reported mood swings** | 1.32 (0.90 – 1.93) | 0.150 |
| **Age** | 0.99 (0.94 – 1.03) | 0.551 |
| **BMI** |  |  |
| **Low / Normal BMI** | Reference | - |
| **High BMI** | 1.19 (0.82 – 1.72) | 0.361 |
| **Education** |  |  |
| **No school/ just primary or high school** | 0.84 (0.49 – 1.43) | 0.519 |
| **Polytechnic or Vocational training** | 0.77 (0.39 – 1.54) | 0.461 |
| **University** | Reference | - |
| **Medical indications for OCs** | 1.11 (0.74 – 1.67) | 0.601 |
| **History of depression** | 1.31 (1.00 – 1.72) | 0.051 |
