# Supplementary material for: Can reporting mood swings during oral contraceptive use predict peripartum depression? Results from the Swedish longitudinal cohort study Mom2B
Source: Eur Psychiatry. 2025 Dec 3;69(1):e4. doi: 10.1192/j.eurpsy.2025.10135 (PMC12816930; doi:10.1192/j.eurpsy.2025.10135)
Supplement: Karaviti et al. supplementary material [file S0924933825101351sup001.zip › S0924933825101351sup021.docx]

|  | Adjusted | Adjusted |
| --- | --- | --- |
| **Variables** | **Odds ratio (95% CI)** | **p value** |
| **Self-reported mood swings** | 1.06 (0.69 – 1.63) | 0.786 |
| **Age** | 1.02 (0.97 – 1.07) | 0.406 |
| **BMI** |  |  |
| **Low / Normal BMI** | Reference | - |
| **High BMI** | 0.83 (0.55 – 1.27) | 0.397 |
| **Education** |  |  |
| **No school/ just primary or high school** | 1.23 (0.69 – 2.19) | 0.476 |
| **Polytechnic or Vocational training** | 1.91 (1.04 – 3.51) | **0.038** |
| **University** | Reference | - |
| **Medical indications for OCs** | 1.83 (1.11 – 3.02) | **0.018** |
| **History of depression** | 2.00 (1.49 – 2.68) | **<0.001** |
